# Mucositis and Peri-Implant Disease Treatment with Chitosan and Titanium Brushes: A Systematic Review

**DOI:** 10.3390/jcm14238306

**Published:** 2025-11-22

**Authors:** Cristian Pappolla Sessa, Adrián Pappolla Sessa, Andrea Martín-Vacas, Cristian Docampo-Vázquez, Juan Manuel Aragoneses

**Affiliations:** 1 Departamento de Medicina y Especialidades Médicas, Facultad de Medicina y Ciencias de la Salud, Universidad de Alcalá de Henares, 28871 Madrid, Spain; cristian.pappolla@edu.uah.es; 2 Facultad de Odontología, Universidad Alfonso X El Sabio, 28691 Madrid, Spain; cdocavaz@uax.es; 3 Private Practice, 45004 Toledo, Spain; 4 Department of Dentistry, Universidad Federico Henriquez y Carvajal, Santo Domingo 10106, Dominican Republic; jmaragoneses@gmail.com; 5 Department of Dentistry, Faculty of Health Sciences, Euneiz University, 01013 Vitoria-Gasteiz, Spain

**Keywords:** peri-implantitis, peri-implant mucositis, implant surface decontamination, titanium brush, chitosan brush

## Abstract

**Background/Objectives:** Peri-implant diseases, including mucositis and peri-implantitis, pose significant clinical challenges due to their inflammatory nature and potential for progressive bone loss. These conditions, primarily driven by bacterial biofilm accumulation, require effective management to prevent further tissue destruction and maintain implant longevity. This systematic review aimed to determine whether the use of chitosan or titanium brushes in the surgical or non-surgical treatment of peri-implant mucositis or peri-implantitis provides clinical advantages—particularly reductions in probing pocket depth (PPD) and bleeding indices—compared to conventional decontamination protocols. **Methods:** The review followed the PRISMA 2020 guidelines and was registered in PROSPERO (CRD42024489556). A comprehensive electronic search was conducted in PubMed, Web of Science, and the Cochrane Central Register of Controlled Trials in December 2024 and updated on 9 November 2025, using predefined Boolean equations. Two authors independently screened studies, extracted data, and assessed the risk of bias using the Cochrane RoB 2 tool. **Results:** The initial and updated searches identified a total of 7470 records, of which nine studies met the inclusion criteria—seven randomized controlled trials and two prospective case series. Among them, five investigated chitosan brushes and three examined titanium brushes. Chitosan brushes showed significant intra-group improvements in PPD and bleeding indices, and titanium brushes demonstrated favorable results in surgical protocols, particularly when combined with regenerative therapies. However, inter-group comparisons revealed no statistically significant superiority of either device over conventional methods. **Conclusions:** Chitosan and titanium brushes are safe and effective tools for implant surface decontamination in peri-implant disease management but do not exhibit clear clinical superiority. The current evidence supports their use as adjunctive devices within standardized treatment protocols. Further large-scale randomized controlled trials with extended follow-up are warranted to strengthen the evidence base and define their clinical role.

## 1. Introduction

The global expansion of implant dentistry has brought forth significant challenges due to the emergence of inflammatory conditions around peri-implant tissues [1,2,3]. These conditions, known as mucositis and peri-implantitis, are characterized by an inflammatory response driven by a significant bacterial component. As such, their treatment focuses on reducing bacterial load and preserving the bone supporting the implant [4]. The 2017 World Workshop defined mucositis as a condition involving erythema, bleeding, and/or suppuration during gentle probing without affecting the supporting bone. When bone loss occurs, the condition is classified as peri-implantitis [1]. Evidence highlights that biofilm accumulation in the peri-implant sulcus is the main etiological factor for mucositis, and its removal and control lead to the resolution of the disease [5,6]. Due to the anatomy of peri-implant tissues, colonization of the sulcus may progress to bone loss, triggering peri-implantitis [1,7].

According to the recent meta-analysis by Reis et al. [8], which applied the 2017 World Workshop diagnostic criteria, peri-implant mucositis affects approximately 63% of patients and 59% of implants, whereas peri-implantitis is present in 25% of patients and 18% of implants. These data confirm that nearly two out of the three individuals with dental implants exhibit peri-implant mucositis, and one in four experience peri-implantitis, underscoring the substantial global burden of these conditions. Despite high implant survival rates, the persistence of such complications highlights the need for standardized preventive and therapeutic protocols, as well as improved methods for implant surface decontamination.

Given the absence of established protocols for treating peri-implantitis and mucositis, the current trend is to align their treatment with that of periodontitis and gingivitis [9,10]. Although the 6th European Workshop on Periodontology concluded that non-surgical treatments for peri-implantitis are ineffective [4], the emergence of new devices, techniques, and evidence prompts the research community not to dismiss non-surgical or combined approaches. Recent literature reviews suggest that certain therapies may be more harmful than beneficial due to the release of particles that can induce osteoclastogenesis. This issue is particularly relevant in abrasive treatments that aim to regularize implant surfaces while debriding tissue, such as high-speed rotary implantoplasty, ultrasonic implant surface treatment, or polishing with pastes and air-powder devices [11,12].

These findings highlight the need to identify methods that can prevent the progression of mucositis to peri-implantitis and treat established peri-implantitis through conservative techniques. In this context, emerging methods include electrolytic cleaning [13], photodynamic therapies such as ErYAG lasers [14,15] or diode lasers [16], ozone therapies [17], ultrasonic oscillating devices made of polyetheretherketone (Vector^®^) [18], antibiotic [19,20,21] and antimicrobial therapies [22], and manual instrumentation with plastic or titanium curettes [23].

While the wide variety of materials and treatments provides therapeutic options, the inability to isolate all factors involved in disease development and the variability of peri-implant defect configurations [24] pose challenges in analyzing available alternatives [25]. Systematic reviews and meta-analyses conducted to date emphasize the lack of consistent evidence supporting any predominant decontamination method as superior to others [9,26,27].

**Figure 1:**
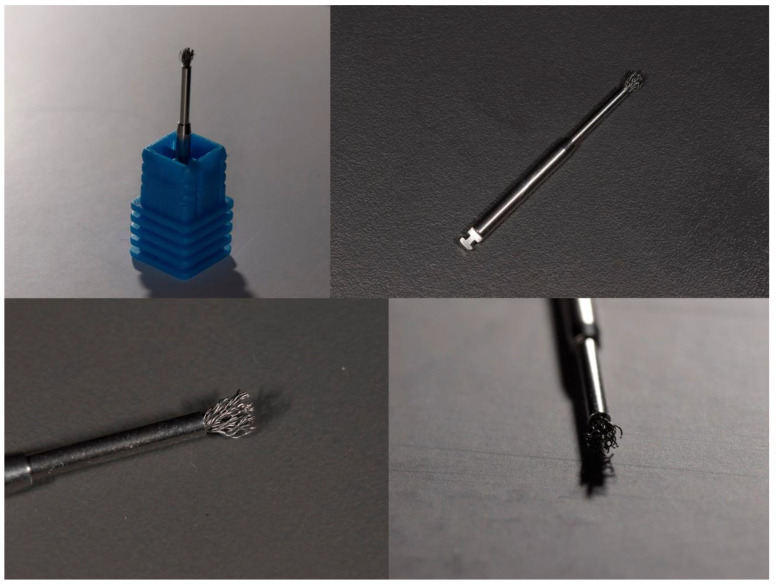
Macrophotographs of a titanium brush (POCKET ^®^ model, Hans Korea, Seoul, Republic of Korea) taken by C.P., showing the device from different angles and magnifications. The images highlight the titanium bristles and brush head morphology used for mechanical debridement of contaminated implant surfaces.

In the last decade, titanium and chitosan brushes have emerged on the market [28,29,30,31] (Figure 1).

### 1.1. Titanium Brushes

First marketed under the name Ti-Brush^®^ (Institut Straumann, AG, Basel, Switzerland), these tools utilize the mechanical properties of titanium to decontaminate implant surfaces without inducing alterations [29]. They consist of a stainless steel shaft and titanium bristles, which clean implant surfaces through rotation or oscillation by mechanical contact [32]. In vitro studies suggest that while NiTi and titanium brushes exhibit high mechanical debridement capacity, they also produce greater surface alterations compared to chemical agents [33,34]. This highlights the need for alternative decontamination tools with different material characteristics, such as chitosan brushes

### 1.2. Chitosan Brushes

Chitosan is a biopolymer derived from chitin extracted from marine crustacean exoskeletons. It has anti-inflammatory properties, no reported allergenic reactions, and proven biocompatibility [35]. Approved for use in surgical dressings as a hemostatic agent, dietary supplements, and topical creams and gels [36,37], chitosan brushes are predominantly marketed under the name LABRIDA BioClean^®^ (LBC, BioClean^®^, LABRIDA AS, Oslo, Norway). These consist of a stainless steel shaft with chitosan bristles, which, like their titanium counterparts, can be used in oscillating or rotational modes. Studies have documented their ability to preserve the roughness of peri-implant surfaces [38].

The objective of this systematic review was to determine whether incorporating chitosan or titanium brushes into surgical or non-surgical protocols for peri-implant mucositis and peri-implantitis provides superior clinical outcomes—particularly regarding reductions in probing pocket depth (PPD) and bleeding indices—when compared with treatment approaches that do not employ these instruments.

## 2. Materials and Methods

This review is reported in accordance with the PRISMA 2020 guidelines [39] (Supplementary Table S1). The protocol was registered in the PROSPERO database (CRD42024489556).

### 2.1. PICOS Framework and Research Question

This review was designed according to the PICO framework to ensure methodological transparency. It focused on patients with peri-implant mucositis or peri-implantitis (Population) who underwent surgical or non-surgical treatment protocols involving chitosan or titanium brushes for implant surface decontamination (Intervention). These approaches were compared with conventional mechanical or adjunctive decontamination methods not including such brushes (Comparison). The primary outcomes (Outcome) were improvements in clinical parameters—specifically reductions in probing pocket depth (PPD), bleeding on probing (BoP), and radiographic bone level (RBL). Accordingly, the research question guiding this review was: “In patients with peri-implant diseases, does the use of chitosan or titanium brushes, compared with standard decontamination techniques, provide greater clinical improvements in PPD, BoP, and RBL?”

### 2.2. Inclusion Criteria

The inclusion criteria focused on patients in good general health requiring surgical or non-surgical treatment for peri-implantitis. The intervention involved the use of titanium or chitosan brushes, either exclusively or in combination, during the treatment. Comparisons were made between protocols incorporating these brushes and those that did not. Outcomes of interest included reductions in probing pocket depth (PPD), bleeding on probing (BoP, mBoP), and suppuration (SoP), along with clinical and relative changes in bone level (BL) and attachment level (CAL/RAL). Patient-reported treatment responses and implant survival rates were also evaluated. Eligible studies were randomized or non-randomized clinical trials involving human participants of any age or gender with implants affected by mucositis or peri-implantitis, provided they included full-text reports with detailed results.in good general health requiring surgical or non-surgical treatment for peri-implantitis.

### 2.3. Exclusion Criteria

The exclusion criteria ruled out studies that were not clinical trials, such as literature reviews, systematic reviews, meta-analyses, conference abstracts, editorials, letters, and opinion pieces. Studies conducted on animals or in vitro settings without direct relevance to human subjects were also excluded. Additionally, articles that did not specifically investigate the use of titanium or chitosan brushes in the treatment of peri-implant diseases, as well as those with inadequate methodological quality, high risk of bias, or insufficiently reported results, were not considered.

### 2.4. Database AMD Search Strategy

A comprehensive electronic search was initially conducted in three major databases—Medline (via PubMed) on 28 November 2024, Web of Science on 15 December 2024, and the Cochrane Central Register of Controlled Trials on 12 December 2024. To ensure the inclusion of the most up-to-date and relevant evidence, this search was repeated and expanded on 9 November 2025. The updated search aimed to identify additional publications released since the initial query and to confirm that no new eligible studies had been overlooked.

All retrieved records were managed using an automated executable code developed by A.P., which facilitated the sorting, identification, and removal of duplicate entries across both search rounds. This tool streamlined the data-cleaning process and ensured consistency and reproducibility of the search results. The final dataset thus represented a unified collection of all unique studies meeting the inclusion criteria, guaranteeing that the literature analyzed in this systematic review reflects the most current state of research available at the time of submission.

A manual search was also conducted in the references of selected studies and in high-impact journals to supplement the bibliography.

The search strategy applied across PubMed, Web of Science, and Cochrane databases is detailed in Table 1. This table illustrates the use of comprehensive Boolean operators that combined keywords related to peri-implant diseases, implant surface decontamination, and the specific devices under investigation, namely titanium and chitosan brushes. The systematic and reproducible approach ensured that potentially relevant studies were retrieved with a high level of sensitivity while minimizing the risk of omitting pertinent evidence.

### 2.5. Study Selection

Two reviewers (C.P. and A.P.) initially screened study titles to determine eligibility based on the inclusion and exclusion criteria. Following agreement on titles, abstracts were reviewed, and relevant studies were selected for full-text analysis and data extraction. Discrepancies in study selection were resolved by a third reviewer (J.M.A.).

### 2.6. Data Extraction and Handling

Two reviewers (C.P. and A.P.) independently collected the data using a standardized, pre-agreed questionnaire designed to capture general study characteristics, including authorship, publication date, inclusion criteria, study design, peri-implantitis diagnosis, sample size, outcomes of interest, follow-up period and other relevant information. Data extracted from the questionnaires were structured in tables, serving as the basis for the risk of bias assessment.

### 2.7. Risk of Bias Assessment

The RoB 2 tool (Revised Cochrane risk-of-bias tool for randomized trials) was used to independently determine the risk of bias for each study by both reviewers (C.P. and A.P.). This tool is a standardized instrument developed by the Cochrane Collaboration to assess the risk of bias in randomized controlled trials. It evaluates five specific domains where bias may be introduced: the randomization process, deviations from intended interventions, missing outcome data, measurement of the outcome, and selection of the reported result. Each domain is judged as having low risk of bias, some concerns, or high risk of bias, based on signaling questions and algorithmic guidance. RoB 2 aims to provide a transparent, consistent, and structured approach to evaluating methodological quality, ultimately informing the certainty of evidence in systematic reviews and meta-analyses. Its application requires a thorough understanding of the study design and reporting to ensure accurate interpretation [40].

### 2.8. Synthesis Methods

A narrative synthesis was undertaken due to the considerable variability in study designs, interventions, and reported outcomes among the included trials. Data were organized thematically according to the type of intervention—surgical or non-surgical—to enable meaningful clinical interpretation. Within each subgroup, the main clinical variables evaluated included probing pocket depth (PPD), bleeding on probing (BoP), and, when available, radiographic bone level (RBL). This thematic approach facilitated comparison between chitosan- and titanium-based protocols while preserving the contextual differences inherent to each treatment modality.

Heterogeneity was assessed qualitatively by two independent reviewers (A.P. and C.P.) through side-by-side comparison of study characteristics summarized in Table 2, Table 3 and Table 4. The reviewers examined differences in diagnostic criteria (peri-implant mucositis versus peri-implantitis), intervention approach (surgical versus non-surgical), adjunctive measures (e.g., regenerative therapy, antiseptic irrigation), follow-up duration, and reporting of clinical outcomes. Discrepancies in interpretation were resolved through discussion and consensus. This evaluation revealed substantial methodological and clinical heterogeneity across studies—particularly regarding disease severity, combination of decontamination methods, and outcome definitions—making statistical pooling inappropriate.

Given these inconsistencies, a structured qualitative synthesis was conducted. Results were integrated descriptively, emphasizing the direction and magnitude of clinical changes rather than inferential statistics. In this synthesis, randomized controlled trials (*n* = 6) and the single prospective non-randomized case series were considered collectively but discussed separately when design differences influenced interpretability. This approach allowed for a transparent integration of evidence, highlighting recurrent trends and contextual factors that may explain the variability in reported effectiveness while acknowledging the methodological diversity of the available data.

### 2.9. Effect Measures

The primary outcome measure across studies was the reduction in probing pocket depth (PPD), while secondary outcomes included reductions in bleeding on probing (BoP), presence of suppuration, changes in radiographic bone levels (RBL), and composite disease resolution metrics. These outcomes were assessed at multiple follow-up intervals ranging from 2 weeks to 18 months. PPD and BoP were consistently measured using calibrated periodontal probes and standardized indices. Most studies reported absolute reductions in millimeters or percentages, with both test and control groups showing improvements over time. However, the magnitude of effect varied widely, and no consistent superiority of either chitosan or titanium brushes was demonstrated. Due to methodological differences and lack of uniform reporting, no pooled effect sizes or standardized mean differences could be calculated. The direction of effect was generally favorable in both groups, but variability in baseline disease severity and adjunctive treatments limited the comparability of outcomes across studies.

### 2.10. Statistical Analysis

Due to the heterogeneity in study designs, intervention protocols, outcome measures, and follow-up durations among the included trials, no formal statistical meta-analysis was performed. Instead, results were synthesized narratively, with emphasis on the direction and consistency of reported clinical outcomes across studies. Descriptive comparisons were made to identify trends in probing pocket depth (PPD), bleeding on probing (BoP), and radiographic bone level (RBL) reductions. Percentual variations and absolute mean changes were extracted where available to support qualitative interpretation. The variability in methodologies, measurement tools, and reporting formats precluded the pooling of data or the calculation of effect sizes. As such, this review does not present inferential statistical comparisons but rather provides a structured analysis of the existing evidence base.

### 2.11. Certainty of Evidence Assesment

The certainty of the evidence was assessed qualitatively based on methodological features of the included studies, such as study design, sample size, risk of bias, and consistency in outcome reporting. Given the narrative nature of this review and the heterogeneity among studies, no formal grading system (e.g., GRADE) was applied. Instead, the reviewers evaluated the overall quality and reliability of the evidence by considering factors such as transparency in methodology, adequacy of follow-up, clarity of outcome definitions, and completeness of reporting. This approach aimed to provide a contextual understanding of the strength of the available evidence without applying a numerical rating system.

## 3. Results

**Figure 2:**
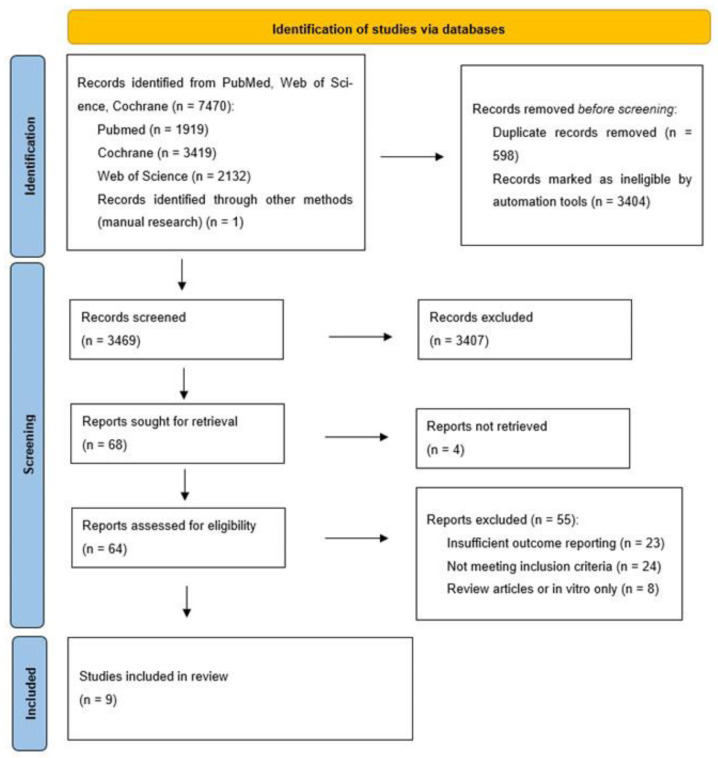
PRISMA 2020 flow diagram illustrating the number of studies identified, screened, assessed for eligibility and included in the systematic review.

The initial electronic search, conducted in December 2024, retrieved a total of 5943 records from the three databases consulted: PubMed (n = 1185), Web of Science (n = 2828), and Cochrane CENTRAL (n = 1929), plus one additional record identified through manual reference screening (Figure 2). After removing 512 duplicates and excluding 2870 records through automated filters, 2561 titles and abstracts were screened. Of these, 2495 were excluded based on relevance criteria. Of the 62 full-text articles assessed for eligibility, 55 were excluded for specific reasons detailed in Figure 2. Twenty-three studies were excluded due to insufficient outcome reporting, as they lacked quantitative data on key clinical variables such as probing pocket depth (PPD), bleeding on probing (BoP), or radiographic bone level (RBL), which prevented their inclusion in the qualitative synthesis. Twenty-four studies did not meet the inclusion criteria established in the review protocol; these primarily involved alternative decontamination modalities unrelated to chitosan or titanium brushes or evaluated peri-implantitis management without specifying disease diagnosis or follow-up parameters. Finally, eight reports were identified as review articles or in vitro studies only and were therefore excluded according to the predefined eligibility criteria restricting the analysis to clinical human studies. These exclusions ensured that only original clinical investigations providing relevant and comparable outcome data were included in the final synthesis. Ultimately, seven studies met all eligibility criteria and were included in the qualitative synthesis.

To ensure that the review remained current, the entire search strategy was repeated on 9 November 2025, using the same predefined Boolean equations across the three databases (PubMed, Web of Science, and Cochrane CENTRAL). This updated search retrieved 7470 records in total: PubMed (n = 1919), Web of Science (n = 2132), and Cochrane CENTRAL (n = 3419). After removing 598 duplicate records, 6873 unique entries remained. Among these, 1527 records corresponded to new publications not included in the 2024 search.

All newly identified records were screened by title and abstract following the original eligibility criteria. As a result, three new clinical studies were deemed eligible and incorporated into qualitative synthesis. These additional studies contributed further data on the use of titanium or chitosan brushes in both surgical and non-surgical management of peri-implant diseases, enhancing the strength and currency of the evidence base.

This update confirms that relevant clinical trials have continued to emerge, and their inclusion reinforces the timeliness, comprehensiveness, and methodological rigor of the present review.

### 3.1. Results of Individual Studies

A total of nine studies met the eligibility criteria and were included in the qualitative synthesis. These comprised six randomized controlled trials (RCTs) and one prospective non-randomized case series. The studies were heterogeneous in terms of design, population size, follow-up duration, and intervention modality. Five of the studies focused on the clinical efficacy of chitosan brushes, while two investigated titanium brushes used either alone or in combination with other surgical protocols. Notably, none of the studies directly compared both brush types within a single trial framework.

Detailed baseline and follow-up measurements of clinical parameters are displayed in Table 4, including probing pocket depth, bleeding indices, and radiographic bone levels. This table provides a more granular view of the outcomes, illustrating consistent intra-group improvements across both chitosan and titanium brush interventions. However, it also underscores the lack of significant inter-group differences, reinforcing the narrative that while both devices are effective in reducing inflammatory signs, neither demonstrated clear superiority over conventional instruments.

Table 2 summarizes the characteristics and main outcomes of studies evaluating non-surgical interventions with oscillating chitosan brushes compared to conventional mechanical debridement techniques. The table highlights variations in sample size, diagnostic criteria, and follow-up duration across trials, while consistently reporting reductions in probing pocket depth and bleeding on probing. Although these studies demonstrated favorable within-group improvements, between-group comparisons generally failed to show statistically significant differences.

Among the chitosan-focused studies, Wohlfahrt et al. [37] conducted a multicenter case series evaluating non-surgical debridement in patients with mild peri-implantitis using oscillating chitosan brushes. The study reported substantial reductions in both probing pocket depth (PPD) and marginal bleeding index (mBoP) over a 6-month follow-up period. In a subsequent RCT by Wohlfahrt et al. [41], the same device was assessed in a split-mouth design comparing chitosan brushes to titanium curettes for the treatment of peri-implant mucositis. Although both groups demonstrated clinical improvement, the differences between them were not statistically significant.

The multicenter randomized controlled trials conducted by Khan et al. [30,42] further explored the non-surgical use of oscillating chitosan brushes versus titanium curettes in patients with mild to moderate peri-implantitis. Both trials reported clinically relevant reductions in PPD and BoP in the test and control groups; however, no statistically significant differences were observed between the interventions. These results suggest that while chitosan brushes may be effective in reducing inflammatory parameters, their advantage over conventional mechanical debridement remains inconclusive in non-surgical contexts.

Table 3 presents the trials that incorporated titanium brushes within surgical protocols, often in combination with regenerative procedures or adjunctive therapies. These studies reported more favorable outcomes in terms of radiographic bone level and probing depth reduction when titanium brushes were used compared with plastic curettes or air-polishing devices. Nonetheless, while the adjunctive effect of titanium brushes appears promising in surgical contexts, the overall heterogeneity of the protocols limits the comparability of results across studies.

Regarding titanium brushes, two studies evaluated their efficacy in surgical settings. De Tapia et al. [28] investigated the adjunctive use of titanium brushes during regenerative surgery for peri-implantitis and observed significant improvements in bone level and clinical outcomes compared to plastic curettes. Toma et al. [23] compared titanium brushes with plastic curettes and air-polishing devices in flap procedures, reporting greater bone preservation in the titanium brush group. However, the overall success rates remained limited across all groups. Koldsland et al. [44] explored the use of chitosan brushes in post-surgical maintenance and found no statistically significant differences when compared to titanium curettes, though both groups maintained clinical stability over 18 months. Overall, individual studies highlighted consistent within-group improvements, yet the between-group comparisons rarely achieved statistical significance, underscoring the need for larger, better-powered trials.

### 3.2. Results of Syntheses

#### 3.2.1. Non-Surgical Protocols

Four clinical studies evaluated the use of an oscillating chitosan brush (LABRIDA BioClean^®^, Straumann, Basel, Switzerland) as part of a non-surgical treatment approach for peri-implant mucositis or mild-to-moderate peri-implantitis [30,37,41,42]. These trials compared chitosan-assisted debridement with conventional mechanical instruments, most often titanium or plastic curettes. Across studies, within-group improvements were consistently observed in bleeding indices (BoP or mBoP) and in probing pocket depth (PPD) reductions ranging from 0.4 to 1.1 mm after 3–12 months of follow-up. However, between-group differences were generally not statistically significant, indicating that the chitosan brush performed comparably to standard debridement. Wohlfahrt et al. [41] also demonstrated that clinical improvements achieved with chitosan brushes could be maintained over one year when combined with regular maintenance visits. Conversely, the studies by Khan et al. [42] highlighted that a proportion of implants continued to exhibit bleeding or suppuration despite therapy, suggesting that disease resolution in non-surgical protocols remains highly dependent on individual plaque control and adherence to maintenance care.

A closer examination of individual trials further supports these observations. In the split-mouth randomized clinical trial by Wohlfahrt et al. [41], both the chitosan brush and titanium curette groups exhibited significant intragroup reductions in BoP and PPD; however, intergroup comparisons revealed no statistically significant differences at any follow-up point. Similarly, Wohlfahrt et al. [37], evaluating patients with mild peri-implantitis, reported parallel improvements in both groups, confirming that the clinical benefits were comparable across instruments. The two randomized studies by Khan et al. [30,42] followed identical non-surgical protocols and yielded congruent findings: measurable reductions in inflammation and pocket depth occurred in both the test (chitosan) and control (titanium curette) arms, yet without demonstrating clear superiority for the oscillating chitosan brush. Collectively, these results indicate that while non-surgical debridement using chitosan brushes is effective and safe, its outcomes remain within the expected range for conventional mechanical decontamination procedures, emphasizing that sustained improvement relies heavily on patient-level plaque control and maintenance adherence.

Recent evidence reinforces the effectiveness of oscillating chitosan brushes as a minimally invasive approach for the management of peri-implant mucositis. In a 2025 randomized clinical trial by Bahçeci et al. [43], 58 patients (103 implants) were treated either with a chitosan brush or with an air-abrasive device using glycine powder. Both groups exhibited significant intragroup reductions in probing pocket depth (PPD), bleeding on probing (BoP), and plaque index after six months. Although between-group differences were not statistically significant, the chitosan brush group demonstrated a slightly faster and greater reduction in PPD (−1.16 mm vs. −0.93 mm) and plaque accumulation (*p* < 0.05). These results confirm that the chitosan brush performs comparably to well-established non-surgical modalities, with potential advantages in plaque control and patient comfort. When integrated with previous findings by Wohlfahrt [37,41] and Khan [30,42], the cumulative evidence suggests that chitosan-based debridement effectively improves soft-tissue health but does not clearly outperform conventional mechanical techniques in the absence of surgical access.

#### 3.2.2. Surgical Protocols

In the surgical intervention group, studies evaluating titanium brushes yielded more favorable clinical outcomes when these devices were used as adjuncts to regenerative or resective procedures. De Tapia et al. [28] demonstrated significant improvements in both PPD and radiographic bone level (RBL) when titanium brushes were incorporated into a surgical protocol that included bone grafting. This suggests a potential synergistic effect when titanium brushes are used alongside regenerative materials. Similarly, Toma et al. [23] found that patients treated with titanium brushes during flap surgery experienced greater bone preservation than those treated with plastic curettes or air-polishing alone, although overall treatment success remained modest across all groups.

The study by Koldsland et al. [44], which focused on the post-surgical maintenance phase, compared chitosan brushes with titanium curettes and observed clinical stability in both arms up to 18 months. While the study confirmed the safety and feasibility of incorporating these devices into supportive therapy, no statistically significant differences were detected between them. Overall, the synthesis indicates that while both chitosan and titanium brushes can contribute to clinical improvement, especially in terms of PPD and BoP reduction, there is insufficient evidence to claim their superiority over conventional methods. The clinical benefit appears to be context-dependent, with slightly more promising results observed when titanium brushes are used within surgical protocols.

More recently, two trials provide consistent evidence that both titanium and chitosan brushes are safe and clinically effective adjuncts to implant surface decontamination. The randomized trial by Park et al. [46] compared rotating titanium brushes with implantoplasty during open-flap surgery in 30 patients. Both interventions yielded significant clinical improvements at 12 months, with mean PPD reductions of 3.6 mm (brush) and 3.3 mm (implantoplasty) and BoP reductions approaching 80%. Notably, radiographic bone levels remained stable in the brush group (Δ0.0 mm ± 0.6), whereas the implantoplasty group experienced an additional bone loss of 0.7 ± 1.2 mm. The procedure was also significantly faster in the brush group (3.0 min vs. 5.5 min, *p* < 0.01). Complementary evidence from the prospective case series by İnce Kuka & Gürsoy [45] demonstrated that incorporating the chitosan brush into a regenerative surgical protocol—together with xenograft (Cerabone^®^, Straumann, Basel, Switzerland) and collagen membrane (Jason^®^, Straumann, Basel, Switzerland)—resulted in substantial improvements in PPD (7.3 → 3.8 mm), BoP (96.9% → 15.6%), and radiographic bone gain (5.5 → 1.4 mm) after 12 months.

### 3.3. Reporting Biases

A formal assessment of reporting bias—such as funnel plot analysis or Egger’s regression test—was not performed in this review due to the absence of pooled data and the limited number of comparable effect sizes across studies. Nonetheless, a qualitative evaluation of potential reporting and methodological biases was conducted to identify patterns of incomplete outcome reporting, heterogeneity, or selective emphasis on favorable results. Several included studies failed to provide full statistical data, such as confidence intervals, standard deviations, or detailed *p*-values, particularly in relation to secondary outcomes like radiographic bone level (RBL) or suppuration. In some non-surgical trials, only partial outcome data were reported across follow-up time points, and baseline comparability between groups was not always fully described. For instance, Wohlfahrt et al. [37] reported reductions in PPD and mBoP but did not present statistical comparisons beyond week 6, whereas Khan et al. [30] provided mean values and standard errors without intergroup effect sizes for key outcomes at interim assessments.

Potential sources of bias were also identified across more recent studies, especially concerning randomization and blinding. Among randomized controlled trials, allocation concealment and examiner masking were inconsistently reported, introducing potential detection bias. The lack of operator blinding was unavoidable in surgical studies employing visible devices, such as titanium or chitosan brushes. Attrition bias was generally low, although Bahçeci et al. [43] reported a moderate dropout rate (≈14%), which may slightly affect precision. In contrast, Park et al. [46] demonstrated strong internal consistency and complete outcome reporting, though the blinding of evaluators was not explicitly stated. The prospective case series by İnce Kuka & Gürsoy [45] presented an inherently higher risk of bias due to the absence of a control group and the use of single-operator outcome assessments.

Although no formal evidence of reporting or publication bias was detected, certain trends warrant cautious interpretation. Most included trials presented favorable or neutral outcomes, and explicitly negative findings were scarce. While this pattern may reflect the genuine clinical effectiveness of the interventions, it also emphasizes the need for transparent reporting regardless of outcome direction. Furthermore, several studies lacked trial registration or predefined protocols, which limited verification of whether all intended outcomes were reported. Adverse events, implant survival rates, and patient-reported outcomes—such as postoperative discomfort or satisfaction—were seldom addressed despite their clinical relevance. Collectively, these limitations highlight the need for greater methodological standardization, preregistration, and comprehensive outcome reporting in future investigations.

### 3.4. Certainty of Evidence

Firstly, although the majority of the studies were randomized controlled trials, some presented moderate risks of bias due to lack of blinding, high attrition rates, or limited reporting of allocation concealment procedures. For instance, not all studies clearly described whether assessors were blinded, which is particularly relevant for outcomes like BoP and PPD that are subject to clinical interpretation. Additionally, variation in intervention protocols and follow-up durations contributed to inconsistency across trials, making it challenging to draw firm conclusions about the relative effectiveness of the decontamination strategies.

**Figure 3:**
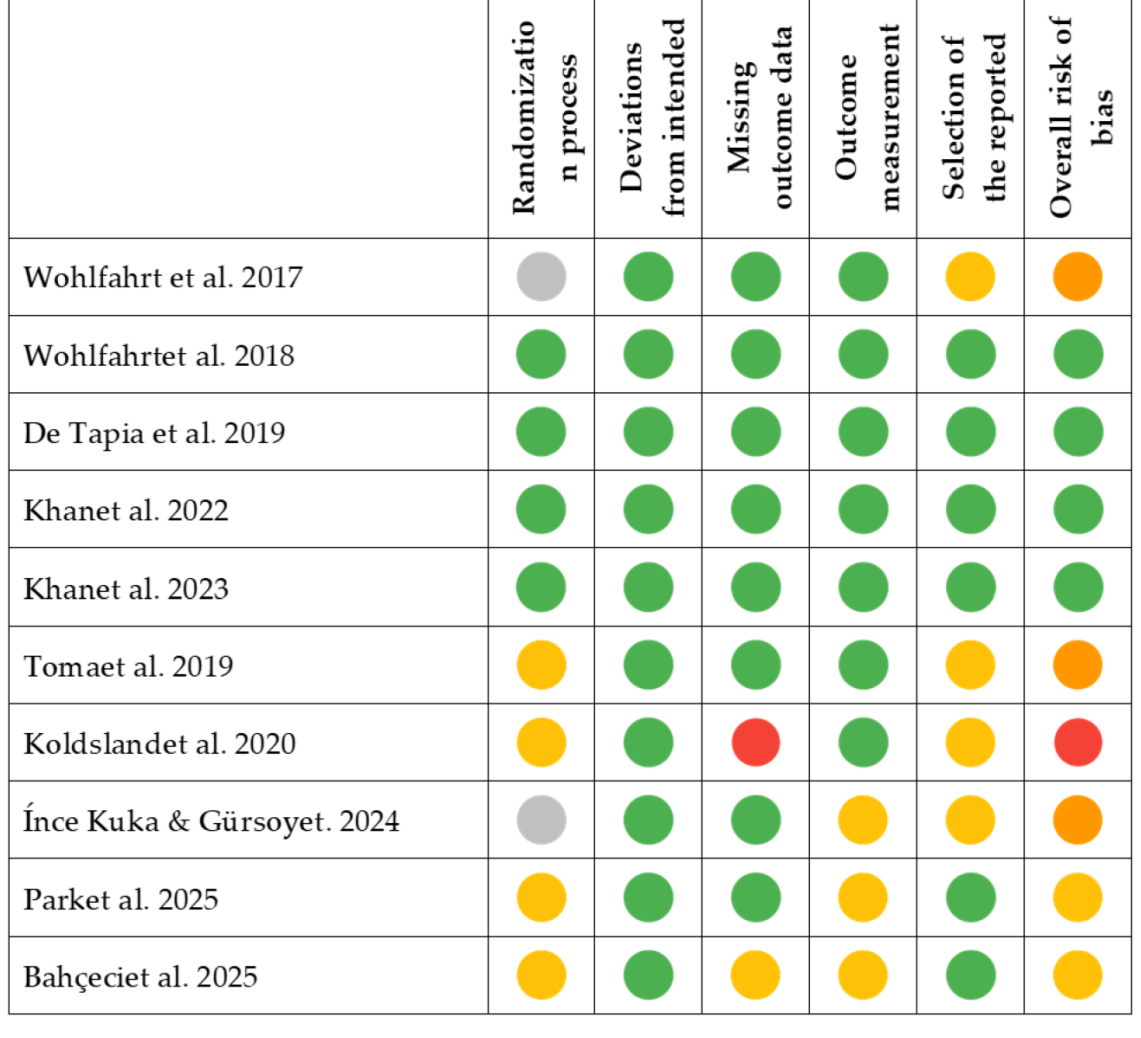
Risk of Bias assessment for included studies using the RoB2 Tool. Symbols: Not applicable (grey); Low risk (green); Some concerns (yellow); Moderate risk (orange); High risk (red) [23,28,30,37,41,42,44,45,46].

Figure 3 reports the assessment of methodological quality and risk of bias for the included studies, conducted using the RoB 2 tool. Most randomized controlled trials were judged to present a low risk of bias across key domains, although some concerns were raised regarding blinding procedures and incomplete outcome reporting. Two studies showed moderate to high overall risk due to missing data or uncertainties in the selection of reported outcomes. These findings highlight the need for more rigorous trial designs to increase confidence in the evidence base.

The precision of the reported outcomes was also limited in several studies, primarily due to small sample sizes and the absence of statistical parameters such as confidence intervals or effect sizes. This made it difficult to assess the robustness of the observed clinical improvements. Despite these limitations, the directness of the evidence was generally high, as all included studies focused on patients diagnosed with peri-implant mucositis or peri-implantitis and evaluated relevant clinical outcomes such as probing pocket depth (PPD), bleeding on probing (BoP), and radiographic bone levels (RBL).

Taken together, the certainty of the evidence supporting the use of chitosan and titanium brushes in the surgical and non-surgical treatment of peri-implant diseases can be considered low to moderate. While individual studies consistently reported within-group clinical improvements, the methodological variability, limited precision, and lack of head-to-head comparisons constrain the strength of the conclusions. Future well-designed, adequately powered randomized trials with standardized protocols and comprehensive reporting are needed to increase confidence in these findings and better define the clinical role of these decontamination tools.

## 4. Discussion

Following Carcuac’s 2020 criteria, the main outcomes considered for this review were the reduction in PPD and BoP, due to their importance as predictive indicators for periodontal and peri-implant diseases [47]. Other factors, such as changes in bone level and elements that might interfere with patient improvement, including antibiotic therapies and oral hygiene instruction, were also considered given their relevance in the available literature [9,48].

### 4.1. Surgical vs. Non-Surgical Comparison

When the findings were stratified according to treatment modality, a distinct pattern emerged between surgical and non-surgical applications. In the non-surgical trials by Wohlfahrt et al. [37,41] and Khan et al. [30,42], which collectively included patients with peri-implant mucositis or mild-to-moderate peri-implantitis, the use of oscillating chitosan brushes consistently produced within-group reductions in bleeding indices and probing pocket depth comparable to those achieved with conventional mechanical debridement. Across these randomized studies, no statistically significant intergroup differences were found, reinforcing that the chitosan brush offers clinical equivalence rather than superiority in non-surgical settings [30,37,41,42].

Conversely, in surgical interventions evaluated by de Tapia et al. [28], Toma et al. [23], and Koldsland & Aass [44], the use of titanium brushes as adjuncts to regenerative or resective procedures was associated with greater reductions in PPD and higher rates of bone fill compared with conventional debridement alone. These improvements were most pronounced when the brush was used in conjunction with regenerative materials, suggesting a mechanical advantage derived from direct access to contaminated implant threads during open-flap surgery. However, complete disease resolution was not consistently achieved, and outcomes varied depending on defect morphology and maintenance adherence.

Taken together, the synthesized evidence supports a context-dependent interpretation of brush-assisted decontamination: chitosan brushes appear most useful in minimally invasive or maintenance protocols, where they match the efficacy of traditional curettes while providing ergonomic advantages, whereas titanium brushes may enhance decontamination in surgical settings that permit full exposure of the implant surface. This conclusion is grounded in the comparative narrative synthesis of both subgroups, highlighting that while these devices are effective and safe, their clinical advantage is conditional on the therapeutic context rather than universally superior.

The collective evidence from non-surgical trials indicates that the use of oscillating chitosan brushes represents a safe and effective mechanical adjunct for the management of peri-implant mucositis and early peri-implantitis. Across multiple randomized clinical trials, including those by Wohlfahrt et al. [37,41], Khan et al. [30,42], and the recent study by Bahçeci et al. [43], consistent reductions in probing pocket depth (PPD) and bleeding on probing (BoP) were observed after treatment, with improvements typically maintained over three to twelve months. Although between-group differences were generally not statistically significant, the chitosan brush achieved clinical outcomes comparable to established mechanical debridement tools such as titanium or plastic curettes and air-abrasive systems. Notably, Bahçeci et al. [43] demonstrated a faster and slightly greater reduction in PPD and plaque index with the chitosan brush compared to glycine air-polishing, suggesting a potential advantage in the early resolution of mucosal inflammation.

Despite these findings, disease resolution remained incomplete in some implants, and a proportion of sites continued to exhibit bleeding or suppuration after treatment, underscoring the limitations of non-surgical therapy for advanced peri-implant lesions. The collective data therefore support the use of chitosan brushes as a minimally invasive and biocompatible adjunct in mucositis or mild peri-implantitis, while emphasizing that long-term stability depends strongly on supportive maintenance and patient compliance.

Within surgical protocols, both titanium and chitosan brushes have demonstrated favorable clinical and radiographic outcomes as adjuncts to surface decontamination. The recent randomized controlled trial by Park et al. [46] compared rotating titanium brushes with implantoplasty during open-flap debridement and showed that both interventions achieved substantial improvements in PPD (−3.6 mm and −3.3 mm, respectively) and BoP (≈80% reduction) after twelve months. Importantly, the titanium brush maintained stable bone levels (Δ0.0 ± 0.6 mm), whereas the implantoplasty group exhibited minor additional bone loss (−0.7 ± 1.2 mm), suggesting that excessive surface flattening may not confer superior biological outcomes. Additionally, the brush significantly reduced operative time, indicating procedural efficiency without compromising effectiveness.

Complementary evidence from İnce Kuka & Gürsoy [45] expanded the application of chitosan brushes into regenerative contexts, demonstrating that when combined with guided bone regeneration (Cerabone^®^ xenograft and Jason^®^ collagen membrane), marked clinical and radiographic gains were achieved after one year, with PPD reduced by 3.5 mm, BoP by 80%, and radiographic bone gain of approximately 4 mm. Collectively, these results suggest that mechanical decontamination using titanium or chitosan brushes provides predictable clinical improvement, particularly when integrated into surgical protocols with regenerative materials. Although direct comparisons remain scarce, current evidence indicates that brushes may offer similar or greater efficacy than traditional instruments such as curettes, implantoplasty, or air-abrasion, while being less invasive and more time-efficient.

### 4.2. Reduction in PPD

In Khan’s 2022 study [30], a significant reduction in PPD was observed following non-surgical debridement using an oscillating chitosan brush, demonstrating considerable clinical improvement. This finding is consistent with their subsequent work [42], which also reported PPD improvements using similar techniques. However, both studies present a high risk of bias due to a lack of blinding and allocation concealment. Similarly, Tapia et al. [28] compared different surgical techniques and found that surgical therapy—comprising surgical debridement with titanium brushes to decontaminate the implant surface followed by regeneration with an alloplastic graft—resulted in significantly greater PPD reductions compared to non-surgical treatment.

The combination of techniques and decontamination methods may also influence PPD values. Other clinical trials, such as Cha’s 2019 study [49], demonstrated that using local minocycline in addition to peri-implant surface decontamination with titanium curettes, ultrasonics, and titanium brushes (manufactured by Dentium^®^) further enhanced PPD reduction. Similarly, Derks et al. [2] found that non-surgical treatments alone were less effective in reducing PPD compared to surgical interventions, suggesting that combined therapies may benefit patients with advanced peri-implantitis.

### 4.3. Reduction in BoP

Authors such as Ramanauskaite et al. [50] and Verket et al. [51] pointed out that while non-surgical treatments can reduce BoP in the short term, their long-term effectiveness is limited without adequate follow-up and maintenance therapies. Roccuzzo et al. [52] suggested that combining surgical and non-surgical techniques could provide more sustainable improvements in BoP reduction and peri-implant tissue stabilization.

As seen in Wohlfahrt’s studies [41], devices like chitosan brushes can achieve significant BoP reductions when used as adjuncts in non-surgical maintenance therapy, highlighting the efficacy of non-invasive treatments. This aligns with the results of Koldsland in 2020 [44], though the latter compared chitosan brushes to titanium curettes in maintenance therapy. Other meta-analyses, such as those presented by Berglundh et al. [1], emphasize the importance of maintenance therapy in BoP reduction. However, Verket et al. [51] argue against the efficacy of these devices, citing a low treatment success rate (12.5%), even while acknowledging BoP reduction.

Regarding surgical therapies, Koldsland et al. [44] demonstrated that surgical treatment combined with chitosan brushes and local antibiotics resulted in significant BoP reduction. Sanz et al. [10] supported these findings, showing that surgical procedures effectively control inflammation when combined with adequate maintenance. These BoP reductions are comparable to those observed by Schwarz et al. [27] using lasers for implant surface decontamination through photodynamic therapy.

### 4.4. Effectivenes of Antimicrobial Therapies

In vitro experiments have shown that oscillating chitosan brushes can achieve a level of biofilm and deposit removal comparable to other implant decontamination modalities, including Er:YAG laser and conventional curettes, while causing less surface alteration. However, Er:YAG laser is considered an independent decontamination approach rather than a purely antimicrobial therapy, and the clinical translatability of these in vitro findings remains limited. [38]. Promising complementary approaches include chemical therapies, such as local minocycline application [49], photodynamic therapies [14], or systemic antibiotics [9]. The lack of literature combining these methods with other surgical techniques might result from statistical errors. Other authors conclude that given the low certainty of evidence, high risk of bias, and limited research, decontamination therapies cannot be deemed more beneficial than standard procedures (mechanical debridement with or without saline) [53].

### 4.5. Impact of Oral Hygiene

One of the key points emphasized in studies on non-surgical therapy is the importance of oral hygiene in preventing and treating peri-implantitis [30,37]. This conclusion aligns with the consensus from the 2017 Workshop on Peri-implant Diseases and the 6th European Workshop on Periodontology, which stress the critical role of rigorous oral hygiene in preventing disease recurrence [1,4]. Chitosan and titanium brushes can be used for periodic maintenance to complement patients’ oral hygiene routines, as evidenced in Wohlfahrt’s study [37].

Peri-implant mucositis and peri-implantitis are fundamentally biofilm-induced inflammatory conditions like gingivitis and periodontitis [43]. Bacterial plaque accumulation on implant surfaces triggers an inflammatory response in the surrounding mucosa, initiating peri-implant mucositis [4]. Notably, peri-implant mucositis is considered a reversible condition—experimental human studies show that removing the microbial biofilm can reverse the early inflammatory changes [54]. In the absence of effective plaque control, however, mucositis lesions may persist and eventually progress to peri-implantitis, with consequent deeper pocket formation and loss of supporting bone. Early intervention through improved oral hygiene and biofilm removal is therefore critical to prevent the transition from mucositis to destructive peri-implantitis [55].

Given this etiologic role of plaque, patient oral hygiene practices have a profound impact on the treatment and outcomes of peri-implant diseases. Clinical observations indicate that patients with higher plaque indices tend to exhibit more peri-implant inflammation—for example, a high plaque score correlates with a greater incidence of peri-implant mucositis and increased probing depths around implants. Both professional supportive care and daily home care share the goal of biofilm elimination; thus, detailed oral hygiene instructions must be provided, and patients need to understand the importance of meticulous plaque control around their implants [56].

Long-term studies consistently show that regular maintenance and good oral hygiene are key to sustaining peri-implant health after therapy. Supportive peri-implant therapy (professional cleanings at individualized recall intervals) significantly improves clinical parameters like PPD and BoP compared to no maintenance [57].

Over an 11-year prospective follow-up, patients who were regularly compliant with peri-implant maintenance had far fewer complications than those with erratic or no maintenance. The regularly compliant group showed about half the prevalence of mucositis (37% vs. 71%) and only one-third the peri-implantitis rate (11% vs. 38%) compared to irregular compliers. Multivariate analysis in that study identified high plaque index and irregular maintenance as significant risk factors for developing peri-implant disease lesions [58].

Consistent maintenance not only reduces bleeding and inflammation but also helps stabilize crestal bone levels and protect long-term implant success. High-quality evidence from clinical trials, cohort studies, and reviews converges on the finding that diligent patient plaque control and maintenance adherence markedly improve treatment outcomes (PPD reduction, BoP improvement) and limit disease progression (including radiographic bone loss) in both non-surgical and surgical management of peri-implant mucositis and peri-implantitis [5,7,56,57,58].

### 4.6. Limitations and Strengths of the Study

This systematic review presents a comprehensive and up-to-date synthesis of available evidence regarding the use of chitosan and titanium brushes in the management of peri-implant mucositis and peri-implantitis. One of its strengths lies in the rigorous application of PRISMA guidelines and the use of the RoB2 tool for bias assessment, which enhances the methodological transparency and reliability of findings. Additionally, the inclusion of both surgical and non-surgical interventions provides a broader understanding of the clinical utility of these brushes across different treatment modalities. However, the review is not without limitations. The limited number of high-quality randomized controlled trials and the significant heterogeneity in study design, sample sizes, follow-up durations, and outcome reporting precluded quantitative meta-analysis. Furthermore, the absence of direct head-to-head comparisons between titanium and chitosan brushes and the underreporting of patient-centered outcomes (e.g., implant survival, adverse events, or patient satisfaction) constrain the generalizability of the conclusions.

### 4.7. Future Lines of Research

Future research should prioritize well-powered, multicenter randomized controlled trials that directly compare chitosan and titanium brushes under standardized protocols, ideally including both surgical and non-surgical treatment arms. These studies should adopt consistent follow-up periods and clearly defined clinical endpoints—such as probing pocket depth, bleeding on probing, and radiographic bone level—while also incorporating patient-reported outcomes and safety data. Moreover, exploring the synergistic potential of these brushes when combined with adjunctive therapies (e.g., local antimicrobials, photodynamic therapy, or regenerative procedures) could help define optimal treatment strategies for peri-implant diseases. Long-term evaluations assessing treatment stability and recurrence rates would be crucial to determine the sustained efficacy and clinical value of these decontamination tools in peri-implant care.

## 5. Conclusions

Chitosan and titanium brushes can effectively reduce probing pocket depth and bleeding in the treatment of peri-implantitis, especially in short-term non-surgical interventions. They have proven to be safe and effective for treating peri-implant pathologies, though without apparent advantages over other techniques and materials. Combining surgical techniques with these devices may be highly beneficial, but treatment choices should be tailored to the severity of peri-implantitis and the patient’s specific characteristics. Further research with larger sample sizes and longer follow-up periods is needed to gain a more comprehensive understanding of the utility of these brushes.

## Figures and Tables

**Table 1:** Search strategy for each database.

Database	Date	Search Strategy
Medline (via PUBMED)	9 November 2025	(“dental implants”) AND (“periimplantitis” OR “peri implantitis” OR “peri-implantitis” OR “implant disease” OR “peri implant disease” OR “peri-implant disease” OR “mucositis” OR “titanium brush” OR “TiBrush” OR “Chitosan brush” OR “Labrida” OR “Labrida Bioclean” OR “therapy” OR “treatment” OR “decontamination” OR “debridement” OR “surface decontamination” OR “pocket depth reduction” OR “bleeding on probing” OR “BoP”).
Web of Science	9 November 2025	TS = (“dental implants”) AND TS = (“periimplantitis” OR “peri implantitis” OR “peri-implantitis” OR “implant disease” OR “peri implant disease” OR “peri-implant disease” OR “mucositis” OR “titanium brush” OR “TiBrush” OR “chitosan brush” OR “Labrida” OR “Labrida Bioclean” OR “therapy” OR “treatment” OR “decontamination” OR “debridement” OR “surface decontamination” OR “pocket depth reduction” OR “bleeding on probing” OR “BoP”)
Cochrane	9 November 2025	(“dental implants”) AND (“periimplantitis” OR “peri implantitis” OR “peri-implantitis” OR “implant disease” OR “peri implant disease” OR “peri-implant disease” OR “mucositis” OR “titanium brush” OR “TiBrush” OR “chitosan brush” OR “Labrida” OR “Labrida Bioclean” OR “therapy” OR “treatment” OR “decontamination” OR “debridement” OR “surface decontamination” OR “pocket depth reduction” OR “bleeding on probing” OR “BoP”)

**Table 2:** Data extraction based on protocol form—Studies with non-surgical techniques. Abbreviations: RCT, randomized controlled trial; OCB: Oscillating Chitosan Brush; PPD, Probing Pocket Depth; BoP, Bleeding on Probing; TC, Titanium Curettes; PI, Plaque Index.

Study	Study Design	Sample Size		Diagnosis (Clinical Signs)	Intervention	Material	Combination		Follow-Up	Results
		Patients	Implants				with Chemical Agents	with Other Techniques		
Wohlfahrt, 2017 [37]	Prospective, non-randomized case series	63	63	Mild peri-implantitis defined as: 1–2 mm bone loss, ≥4 mm PPD, and bleeding on probing	Debridement with OCB at baseline and 3 months	LABRIDA BioClean™ (Straumann, Basel Switzerland)	Not specified. Saline used during debridement.	Not combined with other techniques	2 weeks, 4 weeks, 3 months, 6 months	Significant reduction in BoP and PPD, except between weeks 2 and 4.
Wohlfahrt, 2018 [41]	Multicenter RCT, split-mouth design	11 (13 enrolled, 2 excluded)	24 (12 test, 12 control)	Peri-implant mucositis: ≥4 mm probing depth and bleeding on probing, with no bone loss on X-rays	Debridement with OCB (test) or TC (control) at baseline and 3 months	LABRIDA BioClean™ (test) (Straumann, Basel Switzerland) or TC (control)	Not specified	Not combined with other techniques	2 weeks, 4 weeks, 6 months	Significant reduction in BoP in both groups, with greater improvement in the test group between weeks 2 and 4.
Khan, 2022 [30]	Multicenter RCT	38 (39 enrolled, 1 excluded)	39 (22 test, 17 control)	Mild to moderate peri-implantitis: 2–4 mm bone loss on X-rays, BoP ≥ 2, and probing depth ≥ 4 mm	Debridement with OCB (test) or TC (control)	LABRIDA BioClean™ (test) (Straumann, Basel Switzerland) or TC (control)	Not specified. Saline used during debridement.	Not combined with other techniques	4 weeks, 3 months, 6 months	No statistically significant differences between groups. Significant reductions in PPD and BoP in both groups.
Khan, 2023 [42]	Multicenter RCT	31 (39 enrolled, 8 excluded)	39 (22 test, 17 control)	Mild to moderate peri-implantitis: 2–4 mm bone loss on X-rays, BoP ≥ 2, and probing depth ≥ 4 mm	Debridement with OCB (test) or TC (control)	LABRIDA BioClean™ (test) (Straumann, Basel Switzerland) or titanium curettes (control)	Not specified. Saline used during debridement.	Not combined with other techniques	4 weeks, 3 months, 6 months, 12 months	Significant reductions in PPD and BoP at 12 months in both groups, with no statistically significant differences between them.
Bahçeci, 2025 [43]	RCT	50 (58 enrolled, 8 dropouts)	103 (53 test, 50 control)	Peri-implant mucositis: bleeding and/or suppuration on probing with PPD ≥ 4 mm and no radiographic bone loss beyond the first implant thread	Debridement with OCB (test) or Air-abrasive device using glycine powder (control)	LABRIDA BioClean™ (test) (Straumann, Basel Switzerland) or EMS Airflow^®^ (control) (EMS dental, Nyon, Suisse)	No adjunctive chemical therapy.	Not combined with other techniques.	6 months	Both groups achieved significant reductions in PPD, BoP, and PI vs. baseline. At 24 weeks, BoP improvements were similar; chitosan brush showed slightly faster and greater reductions in PPD and plaque (*p* < 0.05). No adverse events reported.

**Table 3:** Data extraction based on protocol form—Studies with surgical techniques. Abbreviations: TB, Titanium Brush; OCB, Oscillating Chitosan Brush; PPD, Probing Pocket Depth; BoP, Bleeding on Probing; TC, Titanium Curettes; PC: Plastic Curettes; RBL, Radiographic Bone Loss; GBR: Guided Bone Regeneration; CAL, Clinical Attachment Level.

Study	Study Design	Sample Size		Diagnosis (Clinical Signs)	Intervention	Material	Combination		Follow-Up	Results
		Patients	Implants				with Chemical Agents	with Other Techniques		
De Tapia, 2019 [28]	Multicenter RCT	54 (18 test, 36 control)	54 (18 test, 36 control)	Moderate peri-implantitis: 2–4 mm bone loss, BoP ≥ 2, and probing depth ≥ 5 mm	Flap surgery with ultrasonic debridement. Test group used TB; control used PC and hydrogen peroxide irrigation	TB (test group) or PC (control group)	Hydrogen peroxide in both groups	Bone grafting with alloplastic material in both groups	12 months	Significant reduction in PPD and radiographic bone level in the test group compared to the control group.
Toma, 2019 [23]	Randomized clinical trial	30	30 (10 per group)	Peri-implantitis with ≥2 mm bone loss and probing depth ≥ 5 mm	Flap surgery with surface decontamination using titanium brushes (test), plastic curettes, or air polishing (control)	TB (test group), PC, or Perio-Flow^®^ (control group) (EMS dental, Nyon, Suisse)	None	No adjunctive therapies	12 months	All groups showed reductions in PPD and improvements in CAL. Titanium brushes showed greater bone preservation compared to plastic curettes.
Koldsland, 2020 [44]	Randomized controlled trial	142	142	Peri-implantitis with ≥3 mm probing depth, positive BoP, and RBL	Post-surgical maintenance with either OCB (test) or TC (control)	LABRIDA BioClean™ (test group) (Straumann, Basel Switzerland) or TC (control group)	Not specified	None	6, 12, and 18 months	Both groups demonstrated stable outcomes with no statistically significant differences in PPD or BoP between groups.
İnce Kuka & Gürsoy, 2024 [45]	Prospective clinical case series	9	11	Peri-implantitis: PPD ≥ 5 mm with BoP and radiographic bone loss ≥ 2 mm compared with baseline	Open-flap debridement with OCB for implant surface decontamination combined with GBR	LABRIDA BioClean™, Cerabone^®^, Jason^®^ (Straumann, Basel Switzerland)	Saline irrigation only; no chemical decontaminants or antibiotics reported	GBR with xenograft and collagen membrane	12 months	Significant clinical and radiographic improvement: PPD 7.3 → 3.8 mm (*p* < 0.001); BoP 96.9% → 15.6% (*p* = 0.001); RBL 5.5 → 1.4 mm (*p* = 0.010). 100% implant survival, no complications. Authors conclude that GBR + chitosan brush is effective and safe for complex peri-implant defects.
Park, 2025 [46]	RCT	30	15 (test), 15 (control)	Peri-implantitis: PPD ≥ 5 mm with bleeding/suppuration and radiographic bone loss ≥ 2 mm relative to the most coronal intraosseous contact	TB or implantoplasty with carbide burs and polishing	Dentium, Neobiotech^®^, Seoul, Korea	Saline irrigation only; no systemic or local antimicrobials; identical postoperative maintenance protocol in both groups	No combination with other techniques	12 months	PPD reduced by 3.6 mm (brush) vs. 3.3 mm (implantoplasty); RBL stable in brush group (0.0 mm) vs. −0.7 mm in control. 80% of implants achieved pockets ≤ 5 mm. Surgery was faster with the brush (3.0 min vs. 5.5 min, *p* < 0.01). No adverse events reported.

**Table 4:** Detailed extraction of study results. Abbreviations: TB, Titanium Brush; OCB, Oscillating Chitosan Brush; PPD, Probing Pocket Depth; BoP, Bleeding on Probing; TC, Titanium Curettes; PC: Plastic Curettes; RBL, Radiographic Bone Loss; AP, Air-Polishing.

Study	Sample (Implants)	Intervention	Initial			Final			Follow-Up	Results
			Mean PPD	Mean BI	Mean RBL	Mean PPD	Mean BI	Mean RBL		
Wohlfahrt, 2017 [37]	63	OCB	5.15 (4.97; 5.32)	1.86 (1.78; 1.93)	Not specified	4.35 (3.93; 4.77)	0.76 (0.53; 0.99)	Not reported	24 weeks	Significant reductions in PPD and mBoP
Wohlfahrt, 2018 [41]	24	OCB (Test) vs. TC (Control)	Test: 4.27 ± 1.36 mm Control: 4.29 ± 1.50 mm	Test: 1.54 ± 0.78 Control: 1.35 ± 0.85	Not specified	Test: 4.09 ± 1.68 mm Control: 3.95 ± 1.27 mm	Test: 0.70 ± 0.70 Control: 0.74 ± 0.80	Not reported	24 weeks	No significant reductions in BoP between groups
Khan, 2022 [30]	39	OCB (Test) vs. TC (Control)	OCB: 5.3 ± 0.16 mm TC: 5.5 ± 0.29 mm	OCB: 2.33 ± 0.48 TC: 2.24 ± 0.44	OCB: 2.43 ± 0.51 mm TC: 2.58 ± 0.58 mm	OCB: 4.1 ± 1.2 mm TC: 4.2 ± 1.3 mm	OCB: 1.2 ± 0.8 TC: 1.3 ± 0.9	OCB: 2.4 ± 0.5 mm TC: 2.6 ± 0.6 mm	24 weeks	No significant differences between groups
Khan, 2023 [42]	39	OCB (Test) vs. TC (Control)	OCB: 5.2 ± 1.3 mm TC: 5.1 ± 1.2 mm	OCB: 2.1 ± 0.9 TC: 2.0 ± 1.0	OCB: 2.5 ± 0.6 mm TC: 2.4 ± 0.5 mm	OCB: 4.1 ± 1.2 mm TC: 4.2 ± 1.3 mm	OCB: 1.2 ± 0.8 TC: 1.3 ± 0.9	OCB: 2.6 ± 0.5 mm TC: 2.5 ± 0.6 mm	48 weeks	No significant differences between groups
Tapia, 2019 [28]	30	TB vs. PC with H_2_O_2_	Test: 6.17 ± 0.98 mm Control: 6.16 ± 1.27 mm	100% (both groups)	Not specified	Test: 4.15 ± 0.84 mm Control: 3.91 ± 0.93 mm	Not reported	Test: 2.51 ± 1.21 mm Control: 0.73 ± 1.26 mm	48 weeks	Significant reductions in PPD and increases in RBL for test group
Toma, 2019 [23]	70	TB vs. PC vs. Air-polishing	PC: 5.8 ± 0.8 mm AP: 6.2 ± 0.9 mm TB: 6.0 ± 0.9 mm	PC: 90% AP: 85%, TB: 88%	Not specified	PC: 4.5 ± 1.1 mm AP: 4.7 ± 1.2 mm TB: 4.6 ± 1.1 mm	PC: 45% AP: 40% TB: 38%	PC: 4.3 ± 1.0 mm AP: 4.4 ± 1.1 mm TB: 4.4 ± 1.0 mm	48 weeks	Less bone loss with titanium brush compared to plastic curettes
Koldsland, 2020 [44]	135	OCB (Test) vs. TC (Control)	Test: 5.3 ± 1.4 mm Control: 5.1 ± 1.2 mm	Test: 90.1 ± 4.0% Control:89.5 ± 4.1%	Test: 5.1 ± 1.9 mm Control: 5.0 ± 2.1 mm	Test: 4.4 ± 1.8 mm Control: 4.9 ± 2.1 mm	Test: 85.7 ± 4.4% Control: 84.8 ± 4.4%	Test: 4.4 ± 1.8 mm Control: 4.9 ± 2.1 mm	72 weeks	No significant improvements in either group
Ínce Kuka & Gürsoy, 2024 [45]	11	OCB combined with GBR	7.3 ± 0.8	96.9%	5.5 ± 1.4	3.8 ± 0.7	15.6%	+4.1 mm	48 weeks	Radiographic bone regeneration evident at 12 mo; 100% implant survival.
Bahçeci, 2025 [43]	103	OCB (test) vs. AP (control)	Test: 3.81 ± 0.68 Control: 3.74 ± 0.71	Test: 77.4% Control: 78.8%	Not reported	Test: 2.65 ± 0.55 Control: 2.81 ± 0.59	Test: 26.9% Control: 29.6%	Not reported	24 weeks	Comparable BoP reductions at 6 mo; both effective in inflammation control.
Park, 2025 [46]	30	TB (test) vs. Implantoplasty (control)	Test: 7.0 ± 1.4 Control: 7.2 ± 1.3	Test: 100% Control: 100%	Not reported	Test: 3.4 ± 1.1 Control: 3.9 ± 1.0	Test: 20% Control: 25%	Test: Δ0.0 mm (stable) Control: −0.7 mm	48 weeks	Both groups showed marked PPD reduction; brush group slightly better numerically. Radiographic bone maintained in brush group; slight loss in implantoplasty group.

## Supplementary Materials

The following supporting information can be downloaded at: https://www.mdpi.com/article/10.3390/jcm14238306/s1, Table S1: PRISMA 2020 Checklist. Ref. [59] is listed in the supplementary.

## Abbreviations

The following abbreviations are used in this manuscript:

TB	Titanium Brush
OCB	Oscillating Chitosan Brush
PPD	Probing Pocket Depth
BoP	Bleeding on Probing
mBoP	Modified Bleeding on Probing
TC	Titanium Curettes
PC	Plastic Curettes
RBL	Radiographic Bone Loss
AP	Air-Polishing
CAL	Clinical Attachment Level
GBR	Guided Bone Regeneration
RCT	Randomized Controlled Trial
PI	Plaque Index
